# Assessing Solutions for Resilient Dairy Farming in Europe

**DOI:** 10.3390/ani14202991

**Published:** 2024-10-17

**Authors:** Abele Kuipers, Søren Østergaard, Ralf Loges, Jelle Zijlstra, Valerie Brocard

**Affiliations:** 1 Livestock Research, Wageningen University & Research, 6708 WD Wageningen, The Netherlands; jelle.zijlstra7@gmail.com; 2 Department of Animal and Veterinary Sciences, Aarhus University, 8830 Tjele, Denmark; soren.ostergaard@anivet.au.dk; 3 Department of Animal Nutrition and Feed Science/Organic Agriculture, Kiel University, 24118 Kiel, Germany; rloges@gfo.uni-kiel.de; 4 Institut de l’Elevage, 35650 Le Rheu, France; valerie.brocard@idele.fr

**Keywords:** solutions, practices, techniques, expert analysis, farmer workshops, future, dairy sector

## Abstract

The objective of the EU project Resilience for Dairy (R4D) is to develop and strengthen a self-sustainable Thematic Network on resilient dairy farms in 15 European countries. This article focusses on those solutions (practices and techniques) that are assessed contributing to a resilient dairy farming sector. The opinions of experts, farmers, and stakeholders were collected and scored through surveys and in a series of local workshops. Six key contributing knowledge fields are included: economic and social resilience, technical efficiency, environment, animal welfare and health, and societal perception. Assessing these knowledge fields proved to be a good predictor for measuring resilience. Only the impact fields of animal welfare and health and societal perception overlapped each other in response. This study shows differences in the choice of solutions across Europe. Experts from South and East Europe are more positive about the contribution of solutions to resilience than their colleagues from North and West Europe, except for social life items. Expert and farmer/stakeholder opinions differ regarding several of the solutions. Technical efficiency is a leading strategy. Priority topics of interest are communication with society, renewable energy production, strategic hoof trimming, early detection of diseases, monitoring fertility and health, and calf rearing. Besides resilience, attractiveness and readiness of the solutions were also assessed.

## Simple Summary

The EU represents the second largest milk producer at the global level and has the potential to be a key driver of future economic growth. However, the European dairy sector is facing major challenges. To achieve its potential, growth must be delivered from sustainable production systems, which provide viable incomes and an adequate quality of life to dairy farmers, impact less on the environment, and are valued by society. This study focusses on improving the resilience of the dairy farming systems in Europe. Fifteen EU countries participated. Needs and solutions (practices, techniques, and tools) were inventoried by surveys and by a series of local workshops. The solutions were scored by experts, farmers, and stakeholders from the participating countries. The outcomes highlight the diversity in farmer communities and stakeholders in Europe and the practices and techniques most wanted to contribute to building a more resilient dairy sector in times to come.

## 1. Introduction

The EU represents the second largest milk producer at the global level and has the potential to be a key driver of future economic growth [1]. The dairy sector plays a significant role in the maintenance of the human population in many rural areas thanks to economic activities, such as production, processing, marketing, and technical and economic support, and to their support for the local economy in terms of trade, utilities, agrotourism, and the production of traditional and/or high-quality food products. Dairy farming is also crucial for the provision of key ecosystem services for society: nutrient cycling for crop production, conservation of biodiversity, fixing carbon in the soil, etc.

However, the European dairy sector is facing major challenges [2,3]. To achieve its potential, growth must be delivered from sustainable production systems [4,5], which provide viable incomes and an adequate quality of life to dairy farmers, impact less on the environment, and are valued by consumers and the wider society. These challenges and opportunities have been brought into sharp focus by the ending of milk quotas in the European Union in 2015, which removed regulatory constraints to expansion in milk production. The abolition of milk quotas coupled with a reduction in direct market support has been associated with increased uncertainty in the marketplace, more extreme price volatility, and shifts in relative competitiveness between different milk producing regions of the EU [6,7].

Moreover, the societal demands from citizens and consumers put farmers under pressure as they are questioning their production systems and techniques often through an uninformed lens that is nurtured by social media rather than science. The great challenge for dairy farming is to achieve economic and environmental objectives within the current context of climate change, market trends, and societal demands. This must be performed under a set of EU regulations concerning, among others, Water Quality directives, Nature 2000 areas, and more recently the EU Green Deal.

Several studies dealt with the structure of the cattle and dairy sectors in Europe [8,9,10], Australia [11], New Zealand [12], and worldwide [13] and strategy formulation for individual farms [14,15]. However, few works have been conducted on picturing the route forward to a resilient dairy farming sector.

Darnhofer addressed the framework of resilience as follows [16] (p. 195): “Resilience thinking offers a framework to emphasize dynamics and interdependencies across time, space and domains. It is based on understanding social–ecological systems as complex, and future developments as unpredictable, thus emphasizing adaptive approaches to management”. The global concept of resilience applied to agriculture was also described by Meuwissen et al. [17] (p. 7): “Resilience is a latent property of a system. The concept denotes a potential which is activated—and can be observed—only when a system is hit by stress or shocks. It can thus be understood by learning from past trajectories and discussing future scenarios, and from assessing how actual shocks are dealt with”. Quinlan et al. describe the assessing and measuring of resilience and relate it to “understanding and managing complexity and change” [18] (p. 8). Van Dixhoorn explains the concept of resilience from the viewpoint of a herd of animals or the individual animal health status [19] (p. 7): “good disease resilience can be described as the ability of animals to be minimally affected by challenges that can cause disease and, if affected, to recover quickly”.

The EU project Resilience for Dairy (R4D) deals with the challenges the sector is facing in the future [20]. The overall objective is to develop and strengthen a self-sustainable EU Thematic Network on “resilient and robust dairy farms” designed to stimulate knowledge exchanges and cross-fertilization on the topic of resilience among a wide range of actors and stakeholders. In particular, farmers’ needs and solutions (practices, techniques, and tools) contributing to a resilient dairy farm sector were searched for and discussed. This article focusses on the solutions that were identified and subsequently assessed. Three key criteria were applied in the assessments that were carried out: 1. the contribution to economic and social resilience, 2. the contribution to technical efficiency, and 3. the contribution to improvements in the field of environment, animal welfare and health and society-friendly production systems. Next to these criteria, all solutions were also assessed by stakeholders based on their attractiveness and readiness.

## 2. Materials and Methods

**Figure 1:**
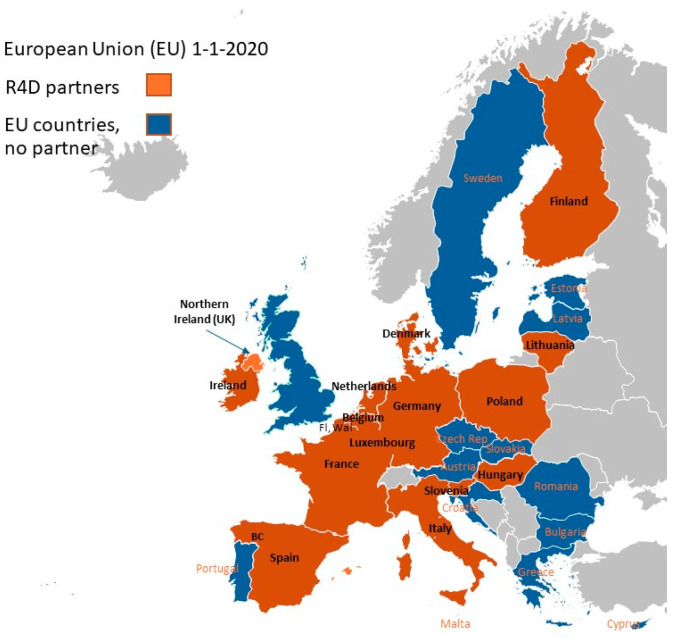
Resilience for Dairy (R4D) partner countries (from UK, only Northern Ireland was included as partner; Belgium had two partners, from Flanders and Wallonia).

The R4D project encompasses 15 EU countries and 16 partners (see Figure 1).

### 2.1. Workflow

**Figure 2:**
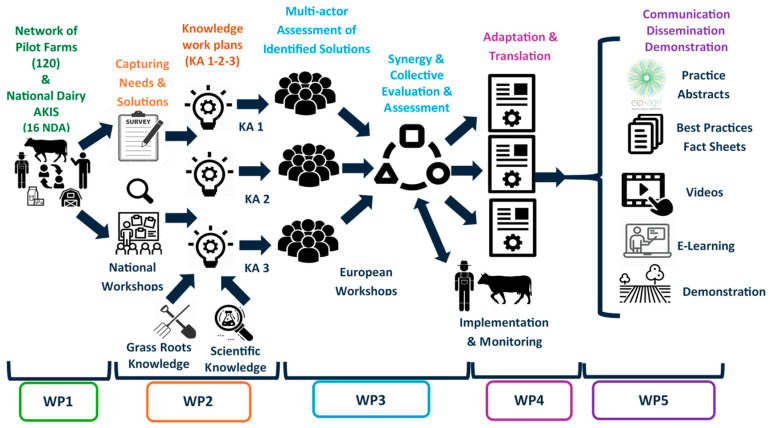
Organization scheme Resilience for Dairy (R4D) (WP1: pilot farms and farmers; WP2: inventory of needs; WP3: assessment of solutions; WP4: monitoring and factsheets; WP5: dissemination).

The workflow of the project is illustrated in Figure 2. The main discussion fora are the international expert meetings and the national meetings of dairy sector stakeholders (NDA workshops, in which NDA stands for National Dairy AKIS, i.e., Agricultural Knowledge and Innovation System). Experts in the key contributing knowledge areas (socio-economics, technical efficiency, and environment/animal health and welfare) were recruited by the R4D-participating universities, research institutes, innovation centers, and extension services. The NDA workshops were held physically, except in some countries when held online a few times during the COVID-19 epidemic. The workshops were organized two times for the assessment of solutions and in total three to six times in each country. The participants in the NDA workshops totaled up to 30 persons including extension and education workers and the R4D pilot farmers in each country (from 4 to 12 farms per country, and thus around 120 farmers in total: for an overview of the farms, follow the Farm Finder link at resilience4dairy.eu) (accessed on 1 August 2024).

Farmer needs were captured in the international experts’ meetings and in the NDA workshops, held in 2021 [21]. Needs were rated from no interest to very interested, by use of a GOOGLE form questionnaire. In total, 535 stakeholders (of which 70% were farmers and 30% other stakeholders) in the 15 participating countries filled in this questionnaire by scoring the pre-printed list of needs. A relatively high number of stakeholders from Belgium participated, because two partners in this country were involved (one from Flanders and one from Wallonia). Missing needs could be added. The results of the questionnaire were discussed in the NDA workshops (one or two per country) and in a European expert workshop, which was organized during a consortium meeting in 2022. These discussions resulted in prioritizing the long list of needs to 43 more widely defined farmer needs, such as work/life balance, income, effective communication, improvement of animal welfare conditions, energy efficiency, and reducing environmental losses. The next step was to make an inventory of solutions, based on the condensed list of needs, that contribute to the three criteria of resilience. These inventories were carried out by all the NDA groups, and resulted in a merged list of 190 solutions, i.e., practices, techniques, and tools. All these solutions were assessed by knowledge experts and the NDA groups in 2022/23 with the aim of choosing solutions that contribute the most to the resilience of the farm business, and some solutions that needed further monitoring to better assess their potential for contributing to resilience.

The final step of the R4D project was to disseminate the accepted solutions in fact sheets and practice abstracts, and as videos.

This article deals with the assessment of the solutions. The 190 solutions were divided in three knowledge areas (KA1, KA2, and KA3), being the three key knowledge fields thought to contribute to a resilient farming system: economic and social resilience, farm technical resilience, and environmental/animal welfare and health resilience.

### 2.2. Assessment of Experts’ Input

**Figure 3:**
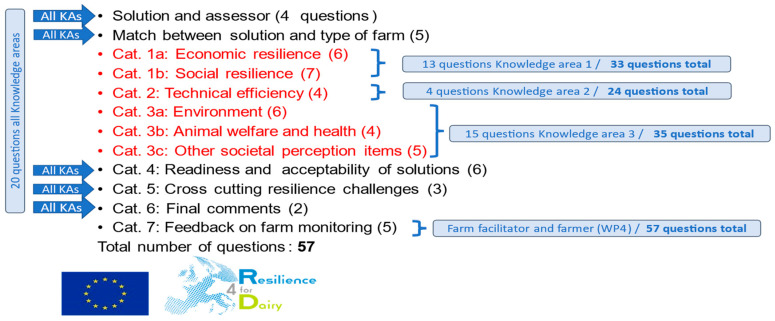
Survey to assess solutions.

Expert assessments of the 190 solutions were organized. An online survey was prepared to evaluate each solution separately. An introduction paragraph was available explaining the use of the survey. The composition of the survey is illustrated in Figure 3. For this study, 32 of the total of 57 survey questions were used (indicated in red color), belonging to knowledge areas KA1, KA2, and KA3. The guideline stated: “To answer the question about the impact of the solution on resilience, take the average dairy farm in your region where this solution would be applicable and attractive as reference to assess the impact you expect”.

All questions had 5 pre-printed answers and an option to answer “no idea”. A total of 66 expert assessors, from 2 to 8 per partner country and about equally split over the three knowledge areas, were selected by the participating partner organizations from the 15 European countries. These experts performed 3329 assessments with a focus on resilience. Thus, on average, about 53 solutions per assessor were obtained. The number of assessments per solution ranged mostly between 15 and 30 evaluations. Solutions with less than 10 assessments were excluded from this study. More attractive solutions were, in general, evaluated more times than less interesting solutions. The expert performed his/her assessments within their area of know-how. Therefore, different groups of experts were involved in the assessments in the three knowledge areas, implying that the comparison of the scores of solutions should preferably be performed within each knowledge area.

**Figure 4:**
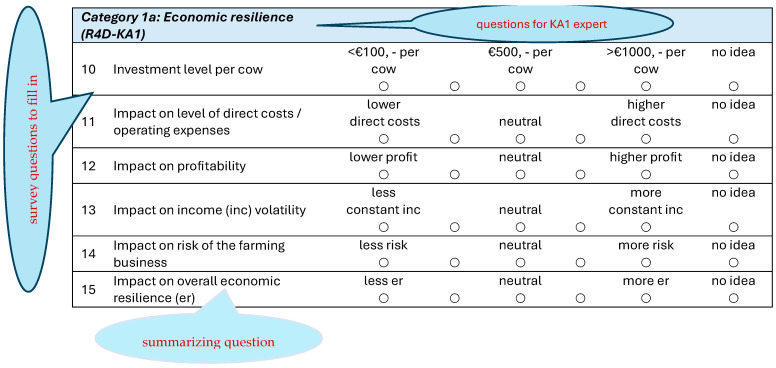
An example of survey questions.

An example of one category of questions, i.e., related to economic resilience, is shown in Figure 4. The answers are ranked from low to high or less to more (or not important to very important) and coded from 1 to 5. However, for some questions, like questions 10 and 14 in Figure 4, the scores had to be reversed because, in general, a low investment and less risk are seen as favorable compared to a high investment and more risk.

The scores are presented in basic statistics, like mean, median, standard deviation, and correlations between scores.

### 2.3. Assessment of NDA Workshops’ Input

Two National Dairy AKIS (NDA) meetings in 15 countries were held to discuss the results of the rounds of assessments of solutions by the experts. The local farm facilitators, who were part of the R4D team, guided the NDA workshops. Those workshops were thoroughly prepared in international and online meetings. Each workshop had up to 30 participants, usually including the R4D pilot farmers and other stakeholders, mostly extension and education workers. The focus of the assessments was on the criteria of attractiveness and readiness for practical application on the farm, but also, the contribution to the resilience of the dairy sector was again included in the evaluation. The NDA group was asked to select the 20 solutions with highest attractiveness. Next, this sample of solutions was scored from 1, least attractive, to 20, most attractive. The same procedure was followed for resilience and readiness for application. The scores were transformed to percentages by dividing the accumulated score of all countries involved through the maximum possible score, expressed by the following equation:

(1) $$Accumulated\;NDA’s\;score\;in\;\%=\left(\sum_{PC1}^{n}\sum_{SC\;SOLi}^{20}/n\times 20\right)\times 100$$

in which PC1 = partner country 1; n = 16 partner countries assessing attractiveness and n = 13 countries assessing resilience and readiness; SC SOLi = score solution i, where i indicates one of the 20 solutions chosen by the NDA; and n × 20 = maximum possible accumulated score.

For some analysis, the NDA groups were split up between North-Western Europe (Scandinavian plus Western countries) and South-Eastern Europa (Mediterranean plus Eastern countries).

A database was prepared to contain all data derived from the experts and those from the NDA workshops.

## 3. Results

The results are divided into the outcomes of the assessment procedure by the experts from 15 countries and the outcomes of the assessment after discussion in the NDA groups.

### 3.1. Experts’ Assessments of Individual Solutions

The results of the experts’ assessments of the 190 solutions are presented separately for the three knowledge areas, i.e., socioeconomics, technical efficiency, and environment/animal welfare and health. Only the results of the assessments by the knowledge area experts for each field are presented (in Table 1, Table 2 and Table 3), because the number of assessments by other knowledge area experts was rather limited for some solutions.

Table 1 shows that the reparceling of land is an urgent need in several countries where history has caused the present farms to be composed of a whole set of small parcels spread over a large area. Lean management, financial management, and knowledge exchange also scored high. Solutions that require additional (family) labor like on-farm milk-processing and fattening of heifers scored low on the social component of resilience.

Table 2 shows a high interest in hoof trimming and calf colostrum management. Monitoring and early detection of diseases, like health and fertility characteristics of cows, as well as a tailored manure application and crossbreeding with beef cattle belonged to the top six choices, albeit with lower scores. Oppositely, tillage to reduce erosion and grazing combined with automatic milking were rated relatively low, although such practices are surely considered of importance in some regions of Europe.

Table 3 shows a great interest in practices related to housing the animals and to improving health and fertility. Biodiversity has become a societal and political topic of attention and is expressed as a challenge to work on. Contrarily, feed additives to reduce rumen methane and dried manure as bedding are considered animal welfare-and-health-unfriendly and are expected to receive a low appreciation from society, although these solutions receive a positive score for environmental resilience.

Some solutions in Table 1, Table 2 and Table 3 have a smaller standard deviation indicating a more similar response, e.g., the need for reparceling of land, using sand as deep bedding in the cubicles, using the solid part of manure as bedding material, exploring farm milk processing, and using feeding additives (blockers) to reduce methane production from cows. These last three solutions received a rather negative response, thus low scores, which may result in less variation.

### 3.2. Experts’ Assessments of Categories of Solutions

**Figure 5:**
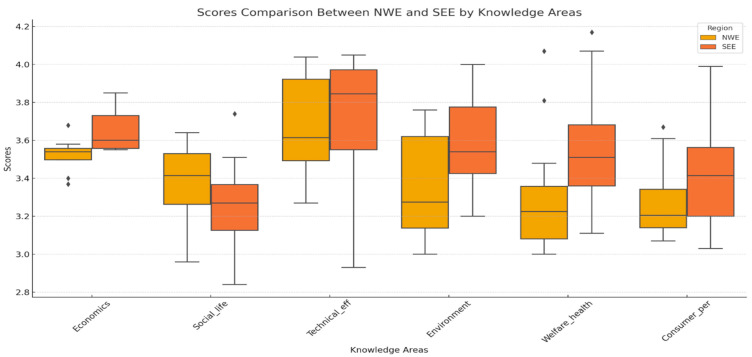
Average scores and spread in scores of categories of solutions per impact field/knowledge area and European region, based on the data from Table 4 (NWE = North and West Europe; SEE = South and East Europe).

The solutions were grouped into 24 categories of solutions, split up between the three knowledge areas. This resulted in a substantial number of assessments per category allowing for more robust results and a division in outcomes for two regions of Europe (see Table 4, column 3). The scores are divided into the scores from experts from North and West Europe (NWE) and those from South and East Europe (SEE). Based on data from Table 4, the 24 categories of solutions are illustrated per impact field in Figure 5.

Table 4 and Figure 5 clearly illustrate the differences in scores assigned by the experts from South and East Europe compared to the experts from North and West Europe. Figure 5 shows that the experts from South-Eastern Europe tended to score, on average, the solutions’ contribution to the various impact fields of resilience somewhat higher than the experts from North-Western Europe. This is especially the case for the scores on the technical efficiency solutions’ categories. Obviously, the experts from South-Eastern Europe expect a more positive effect on the farm business from those practices and techniques, except for social life practices, which are favored by the experts from Western countries.

The impact fields, as illustrated in Table 4 and Figure 5, define six components of resilience. The 190 solutions are assessed and scored for each of the impact fields of resilience. It appeared that the correlations between the scores for the series of solutions between the impact fields were rather low (from −0.15 to +0.25). Only between the scores for the impact fields “technical efficiency” and “animal welfare and health” and, especially, between “animal welfare and health” and “societal perception” were notable correlations found, being +0.30 and +0.57, respectively.

### 3.3. National Dairy AKIS Groups Evaluating Solutions

**Figure 6:**
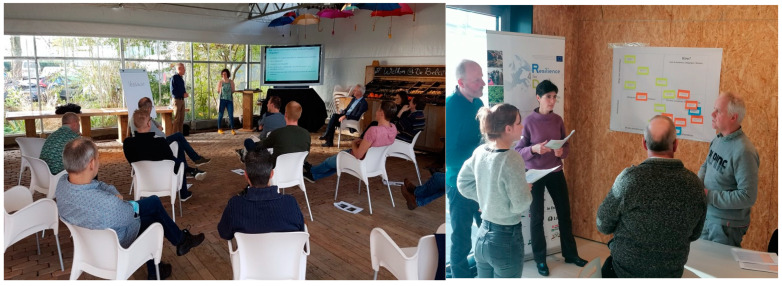
Discussions in stakeholder groups about attractiveness, resilience, and readiness of solutions (Source: R4D).

The three criteria of attractiveness, resilience, and readiness were discussed in the stakeholder (NDA) groups (example of workshops in Figure 6). In each of the NDA workshop in the 15 partner countries (Belgium had two NDA groups), the 20 most preferred solutions were chosen and ranked from 1st to 20th place. Of the total of 190 solutions, 123 solutions were chosen to be discussed at least in one NDA workshop, of which 53 solutions were discussed in only 1 NDA workshop; 21 solutions were discussed in 2 NDA workshops; 23 solutions were discussed in 3 NDA workshops; and 17 solutions were discussed in 4 to 9 NDA workshops. 

**Figure 7:**
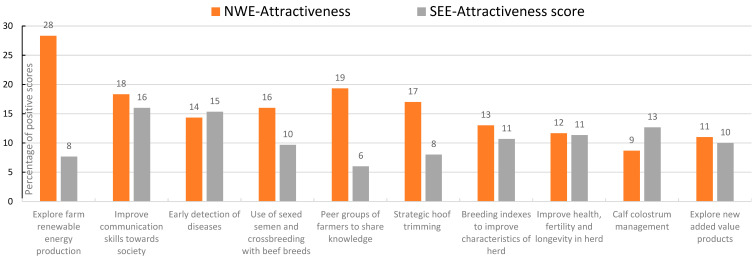
Scoring by stakeholder groups of the 20 solutions with highest attractiveness; this sample of solutions was scored from 1, least attractive, to 20, most attractive; the percentage illustrated in graphic is the accumulated score of all countries involved divided by the maximum possible score (NWE = North and West Europe; SEE = South and East Europe); presented are the 10 solutions with the highest overall scores.

**Figure 8:**
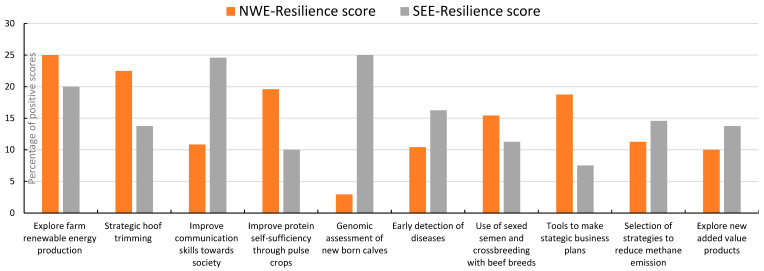
Scoring by stakeholder groups of the 20 solutions with highest contribution to resilience; this sample of solutions was scored from 1, least resilient, to 20, most resilient; the percentage illustrated in graphic is the accumulated score of all countries involved (NWE or SEE) divided by the maximum possible score; presented are the 10 solutions with the highest overall scores.

**Figure 9:**
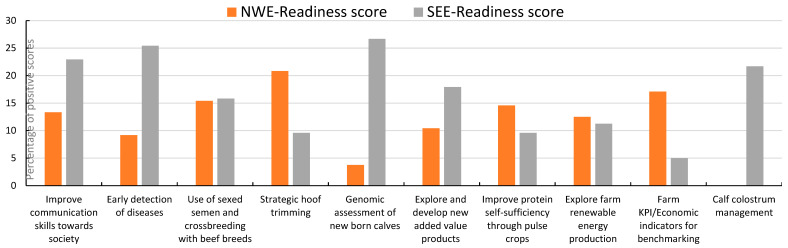
Scoring by stakeholder groups of the chosen 20 solutions most ready for implementation; this sample of solutions was scored from 1, least ready, to 20, most ready for implementation; the percentage illustrated in graphic is the accumulated score of all countries involved (NWE or SEE) divided by the maximum possible score; presented are the 10 solutions with the highest overall scores.

The outcomes of the farmers’ opinions about attractiveness, resilience, and readiness of the solutions are presented in Figure 7, Figure 8 and Figure 9. The outcomes were split up into results from North and West Europe (NWE) and from South and East Europe (SEE).

Exploring the implementation of renewable energy equipment and practices, working with peer groups of farmers and strategic hoof trimming were more targeted as attractive activities by the groups of farmers and stakeholders from North and West Europe than by the farmers from South and East Europe. The improvement of communication skills and the genomic assessment of calves were thought to contribute more to the farm and farm resilience by the farmers in South and East Europe than those in North and West Europe, while strategic hoof trimming, increasing the on-farm protein self-sufficiency, and tools to prepare strategic business plans were higher scored by farmers from North and West Europe. Early detection of diseases, colostrum management, and genomics receive a high readiness level from the farmers from South and East Europe.

## 4. Discussion

This study was about resilience in dairy farming. Resilience is a wide concept. Soubry et al. stated that the concept of resilience can be differently defined by parties, and added that “*though discourse sometimes overlaps thematically, policymakers and farmers have valid, but largely different conceptions of resilience*” [22] (p. 19). Urruty et al. noted that “*stability, robustness, vulnerability and resilience have been increasingly applied to analyze the agricultural context in order to predict the systems response under changing conditions; the four concepts are distinguished by the nature of the system components and by type of perturbation studied*” [23] (p. 1). Banzhaf et al. studied urban challenges related to changes in climate and environment and highlighted “*promising practices for successful partnering around the implementation of nature-based solutions, which make both urban and rural areas more resilient*” [24] (p. 21).

To have some common vision on the concept of resilience, all NDA groups discussed at the start of the project their view on resilience. Key words formulated to describe resilience were adaptability, recovering capacity, flexibility, anticipating, and stress resistant. The experts assessed the solutions’ contribution to resilient farming on a variety of impact fields related to farming (social aspects, economic aspects, technical efficiency, environment, animal welfare and health, and societal perception), while the participants in the NDA workshops discussed the contributions of solutions to the self-perceived overall resilience of the farm business.

The scores of the solutions did not correlate substantially between the different impact fields. Thus, the impact fields appeared to be well chosen components of resilience. Only “animal health and welfare” correlated substantially with “societal perception” (+0.57), indicating that these two concepts overlapped with each other in assessing resilience.

The collection of opinions and scoring data both from experts from universities and research institutes, and from stakeholders (including the pilot farmers), from 15 EU countries in a variety of settings, resulted in an impressive dataset covering a 3-year period. Assessing 190 solutions provided by experts from 15 countries and discussing those solutions in one or more NDA workshops in those countries appeared to be a challenging process. But the dataset had its limitations. For instance, the procedure resulted in a range of assessments per solution. More-attractive solutions seemed to be assessed more than less-favored practices and techniques. To harmonize the work in the 16 NDAs was also quite a tedious task. Additionally, the facilitators of the NDA workshops possibly affected the choice of solutions in some cases by giving more attention in the meetings to their personal research work and results. As an example, lean management was available as a learning package in the R4D project, which likely contributed to the relatively high scores for this solution. A few facilitators were specialists in calf rearing and animal welfare. In those NDA workshops, it looked like friendly calf management practices were well represented in the set of solutions. This phenomenon is known to be difficult to exclude in human-guided processes [25].

We looked for regional differences. Overall, the experts from South-Eastern Europe gave the solutions on the various impact fields of resilience a higher score than the experts from the North-Western region of Europe. It may be that those practices are still in reserve to be applied in the South-Eastern regions, while in the North-Western regions, several of those practices may have already become part of the daily management of the herd and farm. Also noticeable was the perceived relatively high impact of automation, information, and communication technology (ICT), breeding, feeding and health on resilience, as expected by the experts from South-Eastern Europe (see Table 4). Oppositely, these South-Eastern technical oriented experts scored very low on the biodiversity solution. It seems that in their perception, an increasing biodiversity presence on the farm does not fit with an economically efficiently run farm business. This may be reflecting the opinion of a larger group of farmers throughout Europe, when observing the opposition towards the introduction of the EU Green Deal plans. However, the environmentally oriented experts from the South and East and the North and West did not deviate much in their opinion about the inclusion of biodiversity.

It is also somewhat unexpected to see solutions like genomics considered more ready for implementation by stakeholders in the South-Eastern part of Europe, while such technologies and practices are in common use in West Europe. It may be linked to the urgency felt in South-Eastern Europe to start applying these practices more intensively.

The choice of the most attractive solutions in the 16 NDA workshops was repeated up to the end of the project period after a two-year run. The choice of the most attractive solutions did not differ substantially in most countries from the previous inventory. This outcome differed from past studies in which socio-economic opinions did appear to change with time and with the economic and public climate [26]. In particular, the level of the milk price appeared to be a dominant factor in the mindset of farmers in a five-year study [7]. But the two-year period of this study was rather short to experience significant changes. Nevertheless, a change in the choice of solutions was the case for some of the countries. For instance, in Slovenia, the recent public discussions about the introduction of an animal welfare police force, linked to a non-governmental organization, caused much more focus on solutions related to communication with society and animal welfare. In Denmark, there was an intense political debate on implementing a greenhouse gas (GHG) tax on agricultural emissions. Dairy farmers and stakeholders are particularly concerned due to the high CO_2_ levels associated with enteric methane emissions from cattle. This issue became a significant topic of discussion in the later phase of the project period. In the Netherlands, the public discussion about the role of the dairy sector in the environmental domain, especially about the management of manure, caused the NDA group in the most recent workshop to focus much more on soil health, and the proposal even arose to include characteristics of soil health into the nutrient management tools.

The choices of most favorable solutions contributing to a resilient dairy sector differed partly between the experts and the NDA stakeholder groups. Such deviating opinions are quite well known; e.g., Erjavec and Klopcic [27] reported that conventional farmers and stakeholders differed in their opinion from organic farmers and consumers about animal and environmental aspects related to the housing of cattle. Our farmer/stakeholder group was not large enough to be split up into various sub-groups for analysis.

In our study, the farmers and stakeholders gave focus to practices like exploring renewable energy production, improving communication with society, exploring added value products, and using sexed semen and cross breeding with beef breeds, while the experts opted more for financial and lean management, manure application precision techniques, barns for more animal welfare, and biodiversity implementation. Technical practices and techniques concerning strategic hoof trimming, early detection of diseases, monitoring of health and fertility, and calf rearing were prioritized by experts as well as stakeholders. Practices, techniques, and tools for improving the efficiency of the herd and farm usually dominated the discussions.

## 5. Concluding Remarks

It was a challenging process to collect and assess the series of solutions from the 15 participating countries by a group of experts and via the National Dairy AKIS workshops.

The choices of solutions were likely affected by the experts participating in the Resilience for Dairy project, and by the facilitation of the workshops, choice of farmers, etc.

The impact fields economic resilience, social resilience, technical efficiency, environment, animal welfare and health, and societal perception appeared to be good predictors for resilience as defined in this project. Only animal welfare and health and societal perception overlapped each other in response.

There are differences in focus on solutions across Europe (especially East versus West); for instance, thoughts about biodiversity differ significantly; in general, experts from South and East Europe are more positive about the contributions of solutions to resilience than their colleagues from North and West Europe.

Expert and farmer/stakeholder opinions appeared not to be the same for several of the solutions; they live in different environments.

Technical efficiency appeared to be a leading strategy at farm level.

Communication with society, renewable energy production, strategic hoof trimming, early detection of diseases in the herd, monitoring fertility and health of the animals, and calf rearing were widely mentioned topics of interest.

## Figures and Tables

**Table 1:** Experts’ choice and scores (from 1 to 5) of the top six scoring and two low scoring ^1^ socio-economic solutions’ impact on resilience (average scores and standard deviations).

Title of Solution	Knowledge Area/Impact Field					
	Economic Resilience		Social Resilience		Economic + Social Resilience	
	Mean	SD	Mean	SD	Mean	SD
Lean management	4.17	0.48	3.70	0.57	3.94	0.41
Reparceling of land	3.86	0.31	4.03	0.41	3.95	0.29
Manage cash flows, investment, and risks to increase mental health and resilience of farmer	4.12	0.30	3.50	0.67	3.81	0.42
Improve quality consultancy services, engage advisory in farm management	3.84	0.48	3.50	0.60	3.65	0.50
Tools to make business plans to support strategic decisions	3.76	0.53	3.53	0.51	3.64	0.39
Peer groups of farmers to share knowledge using facilitation methods	3.78	0.43	3.46	0.42	3.62	0.34
On-farm dairy heifer valorization	3.43	0.59	2.50	0.49	2.96	0.51
Exploring on farm milk-processing	3.20	0.36	2.54	0.38	2.87	0.27

^1^ Low scoring solutions in shaded rows.

**Table 2:** Experts’ choice and score (from 1 to 5) of the top six scoring and two low scoring ^1^ technical efficiency solutions’ impact on resilience (average scores and standard deviations).

Title of Solution	Knowledge Area/Impact Field	
	Technical Efficiency	
	Mean	SD
Strategic hoof trimming	4.72	0.40
Calf colostrum management	4.46	0.60
Sensors monitoring insight in health and fertility	4.17	0.54
Manure application tailored to needs plant	4.13	0.82
Early detection of diseases	4.11	0.59
Cross-breeding with beef cattle	4.06	0.80
Conservation tillage to reduce erosion	3.36	1.01
Combining efficient grazing with robotic milking	3.13	0.74

^1^ Low scoring solutions in shaded rows.

**Table 3:** Experts’ choice and score (from 1 to 5) of the top six scoring and two low scoring ^1^ environmental/animal welfare and health solutions’ impact on resilience (average scores and standard deviations).

Title of Solution	Knowledge Area/Impact Field							
	Environment		Animal Welfare and Health		Societal Perception		Welfare and Health + Environment + Perception	
	Mean	SD	Mean	SD	Mean	SD	Mean	SD
Improvement of health, fertility and longevity in herds	3.41	0.40	4.40	0.62	3.88	0.46	3.90	0.44
Freewalk farming system	3.68	0.56	4.26	0.51	3.54	0.46	3.84	0.41
Agroforestry on dairy farms	3.81	0.44	3.46	0.57	3.74	0.39	3.67	0.38
Barns for more animal welfare with access to outside	3.15	0.43	4.31	0.51	3.55	0.50	3.67	0.32
Biodiversity implementation package for dairy farms	3.94	0.41	3.38	0.55	3.43	0.50	3.60	0.27
Apply sand as deep bedding in cubicles to improve health, welfare and productivity	3.20	0.30	4.12	0.56	3.48	0.36	3.60	0.31
Use solid part of slurry as bedding material in cubicles	3.59	0.35	3.20	0.27	2.91	0.24	3.22	0.18
Feed additives to reduce rumen methane production	3.51	0.42	2.96	0.54	3.00	0.32	3.17	0.26

^1^ Low scoring solutions in shaded rows.

**Table 4:** Average scores for categories of solutions related to six impact fields, belonging to the knowledge areas socio-economics, environment/animal welfare and health, and technical efficiency ^1,2^.

Category of Solutions	No. of Solutions	No. of Assessments		Assessors from the Three Knowledge Areas’ Resilience Scores for Solutions Related to the Impact Field:									
				Economics		Social Life		Environment		Welfare and Health		Societal Perception	
**Socio-Economics**		**NWE**	**SEE**	**NWE**	**SEE**	**NWE**	**SEE**	**NWE**	**SEE**	**NWE**	**SEE**	**NWE**	**SEE**
Business	9	121	82	3.55	3.55	3.12	3.08	3.17	3.41	3.15	3.53	3.33	3.51
Collaboration	5	54	43	3.53	3.55	3.52	3.51	3.37	3.51	3.20	3.41	3.21	3.23
Dairy policy	4	36	35	3.55	3.85	3.31	3.14	3.16	3.20	3.31	3.45	3.32	3.20
Education	9	98	67	3.53	3.61	3.38	3.30	3.21	3.54	3.44	3.79	3.38	3.81
Interest in farming	5	45	40	3.68	3.69	3.45	3.24	3.05	3.46	3.15	3.33	3.24	3.46
Labor efficiency	4	46	30	3.58	3.85	3.56	3.32	3.23	3.53	3.32	3.69	3.21	3.40
Social welfare	8	76	55	3.37	3.56	3.64	3.74	3.08	3.29	3.09	3.37	3.11	3.18
Added value products	2	26	23	3.40	3.59	2.96	2.84	3.60	3.84	3.25	3.63	3.67	3.64
**Environment/anim. health and welfare**						**Technical efficiency**							
Animal welfare	12	145	108			3.33	3.98	3.11	3.30	4.07	4.07	3.47	3.43
Biodiversity	4	59	32			3.67	2.93	3.75	4.00	3.48	3.42	3.43	3.32
Carbon footprint	5	75	37			3.56	3.51	3.65	3.77	3.00	3.13	3.16	3.08
Energy	3	43	24			3.67	3.55	3.50	3.63	3.00	3.29	3.18	3.03
Manure management	9	71	54			3.27	3.88	3.65	3.83	3.04	3.15	3.10	3.14
Emissions	3	23	23			4.00	3.97	3.76	3.80	3.05	3.38	3.20	3.20
Society	2	22	24			3.50	3.98	3.00	3.20	3.00	3.29	3.61	3.48
Water	3	26	23			4.00	3.53	3.66	3.43	3.10	3.49	3.19	3.08
**Technical efficiency**													
Automation	1	15	17			3.50	3.87	3.08	3.60	3.44	4.17	3.18	3.25
Breeding	11	136	93			3.86	4.01	3.27	3.55	3.35	3.68	3.14	3.76
Crops	13	150	78			3.92	3.55	3.75	3.91	3.02	3.11	3.14	3.32
Feeding	22	221	118			3.54	3.84	3.28	3.54	3.20	3.64	3.09	3.57
Grassland	17	197	75			3.47	3.73	3.61	3.79	3.34	3.63	3.27	3.76
Health	23	265	120			3.93	4.05	3.13	3.40	3.81	3.94	3.40	3.99
Herd management	8	98	48			4.04	3.77	3.35	3.55	3.34	3.59	3.09	3.50
Info and communication technology	5	54	41			3.27	3.85	3.14	3.54	3.38	3.92	3.07	3.56

^1^ Number of assessments by the expert groups on some categories were low and excluded (fewer than 10 assessments). This accounts for the assessments by social–economic experts on the environment/animal health and welfare solutions, and on technical efficiency, and those by technical efficiency experts on the socio-economic solutions. ^2^ Shaded areas indicate the scores assigned by the experts about their own knowledge area. Each colour represents a group of experts related to a knowledge area. Moreover, the categorie of solutions also refers to a knowledge area of the same colour.

## Data Availability

The data presented in this study are publicly available upon request.
